# Know Your Audience: Predictors of Success for a Patient-Centered Texting App to Augment Linkage to HIV Care in Rural Uganda

**DOI:** 10.2196/jmir.3859

**Published:** 2015-03-24

**Authors:** Mark J Siedner, Data Santorino, Jessica E Haberer, David R Bangsberg

**Affiliations:** ^1^Center for Global HealthMassachusetts Genneral HospitalHarvard Medical SchoolBoston, MAUnited States; ^2^Department of PediatricsMbarara University of Science and TechnologyMbararaUganda

**Keywords:** telemedicine, text messaging, randomized controlled trial, Uganda, HIV

## Abstract

**Background:**

Despite investments in infrastructure and evidence for high acceptability, few mHealth interventions have been implemented in sub-Saharan Africa.

**Objective:**

We sought to (1) identify predictors of uptake of an mHealth application for a low-literacy population of people living with HIV (PLWH) in rural Uganda and (2) evaluate the efficacy of various short message service (SMS) text message formats to optimize the balance between confidentiality and accessibility.

**Methods:**

The trial evaluated the efficacy of a SMS text messaging app to notify PLWH of their laboratory results and request return to care for those with abnormal test results. Participants with a normal laboratory result received a single SMS text message indicating results were normal. Participants with an abnormal test result were randomized to 1 of 3 message formats designed to evaluate trade-offs between clarity and privacy: (1) an SMS text message that stated results were abnormal and requested return to clinic (“direct”), (2) the same message protected by a 4-digit PIN code (“PIN”), and (3) the message “ABCDEFG” explained at enrollment to indicate abnormal results (“coded”). Outcomes of interest were (1) self-reported receipt of the SMS text message, (2) accurate identification of the message, and (3) return to care within 7 days (for abnormal results) or on the date of the scheduled appointment (for normal results). We fit regression models for each outcome with the following explanatory variables: sociodemographic characteristics, CD4 count result, ability to read a complete sentence, ability to access a test message on enrollment, and format of SMS text message.

**Results:**

Seventy-two percent (234/385) of participants successfully receiving a message, 87.6% (219/250) correctly identified the message format, and 60.8% (234/385) returned to clinic at the requested time. Among participants with abnormal tests results (138/385, 35.8%), the strongest predictors of reported message receipt were the ability to read a complete sentence and a demonstrated ability to access a test message on enrollment. Participants with an abnormal result who could read a complete sentence were also more likely to accurately identify the message format (AOR 4.54, 95% CI 1.42-14.47, *P*=.01) and return to clinic appropriately (AOR 3.81, 95% CI 1.61-9.03, *P*=.002). Those who were sent a PIN-protected message were less likely to identify the message (AOR 0.11, 95% CI 0.03-0.44, *P*=.002) or return within 7 days (AOR 0.26, 95% CI 0.10-0.66, *P*=.005). Gender, age, and socioeconomic characteristics did not predict any outcomes and there were no differences in outcomes between those receiving direct or coded messages.

**Conclusions:**

Confirmed literacy at the time of enrollment was a robust predictor of SMS text message receipt, identification, and appropriate response for PLWH in rural Uganda. PIN-protected messages reduced odds of clinic return, but coded messages were as effective as direct messages and might augment privacy.

**Trial Registration:**

Clinicaltrials.gov NCT 01579214; https://clinicaltrials.gov/ct2/show/NCT01579214 (Archived by WebCite at http://www.webcitation.org/6Ww8R4sKq).

## Introduction

The promise of mobile phone-based interventions to improve health care delivery in resource-limited settings has been well described [1,2]. The widespread availability of cellular networks coupled with the exponential growth in mobile phone ownership [3] creates an opportunity to leverage limited human resource capacity in resource-limited settings through improved patient-provider communication, information management, clinical decision making, disease surveillance, as well as monitoring and evaluation [2]. Despite a rapid increase in investments in mHealth programs, there are few reports of successfully implemented mHealth interventions in sub-Saharan Africa [4,5].

End-user characteristics that influence acceptance and use patterns of mHealth interventions are critical to successful implementation [6], particularly in settings with variable literacy and technology experience [7,8]. Although there is much literature on the behavioral science of novel technology acceptance and uptake in resource-rich settings [9,10], similar data are largely lacking from resource-limited settings. Data are even sparser on technology acceptability for low-literacy end users. A handful of studies have evaluated the general acceptability of mobile phone-based interventions in these scenarios [11-15], but there is an important need to better understand the barriers and facilitators of technology acceptance for low-literacy populations for whom many mHealth interventions are intended. Of particular importance to patient end users is attention to privacy and confidentiality [11,12,14,16], which can have health and safety implications for stigmatized health conditions, such as human immunodeficiency virus (HIV) infection [17].

We previously reported on the perceived acceptability of a mobile phone app to improve communication of laboratory results to patients at an HIV clinic in southwestern Uganda [12], and on the results of a clinical trial at the same clinic to improve linkage to care through a combination short message service (SMS) text messaging app and transportation reimbursement intervention [18]. In the clinical trial, participants with abnormal results who received SMS text messages about CD4 count results and a transportation reimbursement had significantly improved time to clinic return and time to antiretroviral therapy (ART) initiation than those in a preintervention control period. Here we report results of a prespecified secondary analysis restricted to participants in the intervention period who were sent SMS text messages with the following specific objectives: (1) to identify predictors of self-reported message receipt, accurate identification, and appropriate return to clinic in response to the SMS text messaging app and (2) to evaluate the comparative efficacy of randomly allocated SMS text formats to optimize the balance between confidentiality and accessibility of health-related SMS text communications for low-literacy end users.

## Methods

### Study Setting and Participants

Data for this analysis were collected as part of a randomized clinical trial of an SMS text app to notify people living with HIV (PLWH) of their laboratory results and request return to care for those with abnormal results. Full details and preliminary results of the trial have been reported previously (NCT01579214) [18]. We present the results of a secondary analysis restricted to those who were sent at least one SMS text message as part of the intervention arm of the clinical trial. The goal of the analysis was to identify predictors of receipt, comprehension, and appropriate response to an SMS text-based clinical communication intervention. Patients were eligible for the study if they were actively in care at the adult HIV clinic of the Mbarara Regional Referral Hospital in Western Uganda, had access to a mobile phone, lived in the clinic catchment area, and were undergoing CD4 count testing. Clinicians specified an abnormal test threshold for each participant, defined as a result that would prompt early return to care for treatment initiation, regimen change, or clinical evaluation.

### Intervention Development

We designed the SMS text-based intervention based on a conceptual framework derived from a preliminary survey of clinic patients, conducted to understand barriers to linkage and acceptability of SMS text message-based health communications [12]. We learned that the two most cited patient-reported barriers to clinic return after abnormal laboratory results were lack of efficient communication with clinical staff and difficulty affording the costs of transportation to clinic [19-21]. Based on this input, we designed a combination intervention to address both of these factors through (1) an SMS text-based communication system to inform patients of their laboratory results and (2) a transportation reimbursement for those with abnormal results if they returned within 7 days of the first message. We involved a multidisciplinary team, including research staff, programmers, clinicians, and patients in development of the SMS text intervention. Key considerations included messaging format that balanced privacy and clarity, and optimization of message timing and frequency. We pilot-tested the intervention with study staff prior to study implementation.

### Study Procedures

**Figure 1:**
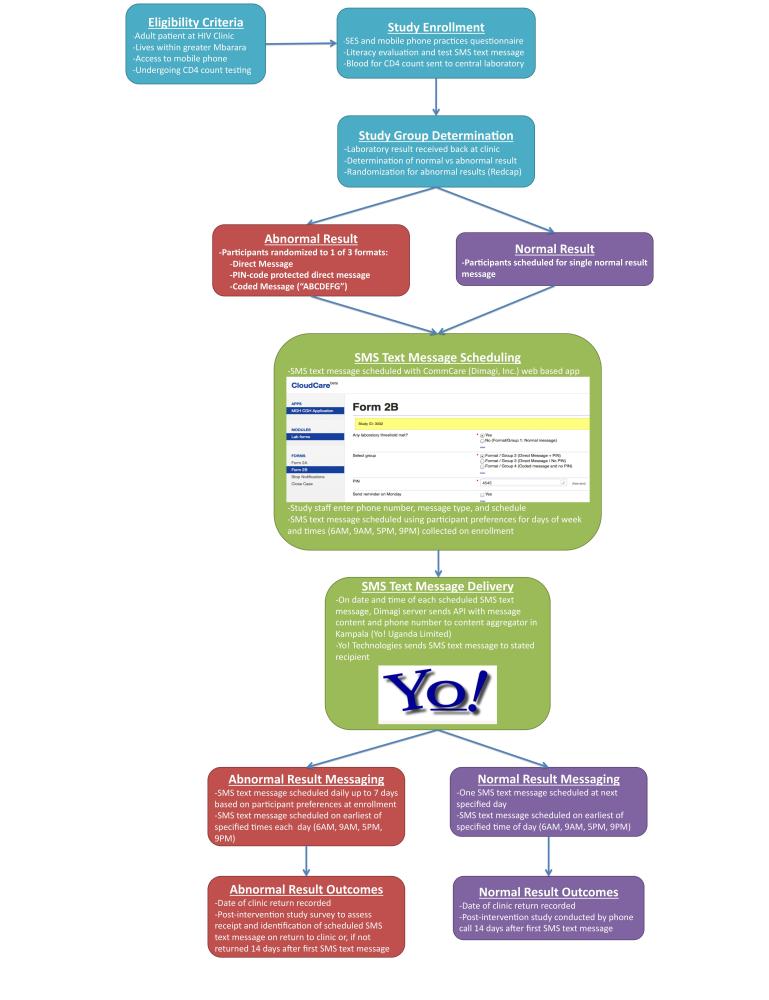
Study schema.

Participants were approached for enrollment after completion of their clinic visit and blood collection for CD4 count testing. Study staff administered a questionnaire on the day of enrollment to collect data on socioeconomic status and mobile phone use characteristics. As part of the survey, participants were asked to read a complete sentence in the local language (Runyankole). For those who had a mobile phone available on the day of enrollment, a test message was sent and participants were asked to open and read the test message (“ABCDEFG”). Finally, preferences for receiving SMS text messages were recorded, with options for day of the week and time of day (options included 6 am, 9 am, 5 pm, and 9 pm). Participants were instructed to return to clinic within 7 days of the first abnormal SMS text message. Those who did return within 7 days received a transportation reimbursement (Figure 1).

### Study Groups

Per standard clinical protocols, blood samples were sent to an offsite central laboratory where they were processed and a laboratory result form was returned to the clinic. On receipt of test results at the clinic, study staff determined whether test results were below the prestated abnormal threshold to classify each participant as having a normal or abnormal test result. Participants with a normal laboratory result received a single message indicating a normal result and requesting return on the date of their next visit (“Your laboratory result was within the normal range. Please return to clinic on your scheduled date”)*.*

Participants with an abnormal result were randomized with the use of the randomization module in Research Electronic Data Capture (REDCap [22]) to 1 of 3 SMS text message formats to evaluate trade-offs between clarity and privacy:

1. Notification that results were abnormal and requesting return to clinic (direct message): “This is an important message from your doctor. You had an abnormal test result. You should return to clinic as soon as possible”
2. An identical message as the direct message, but prompted by an initial requirement to enter a 4-digit personal identification number (PIN message), which was selected by participants on the day of enrollment and given to the patient on a form to take home on the day of enrollment: “Please enter your PIN code to see your message”
3. A coded message which was explained on enrollment to indicate an abnormal test result and signify early return to clinic (coded message): “ABCDEFG”

Participants with abnormal test results were eligible to receive messages daily for up to a maximum of 7 days. The number and timing of messages was determined by their scheduling preferences on enrollment.

### Text Message Scheduling and Transmission

Research staff scheduled messages through a Web-based messaging app (CommCareHQ, Dimgi, Inc, Cambridge, MA, USA) on the date of laboratory result receipt. The CommCare app sent an automated application program interface (API) with the mobile phone number of the participants along with the message content to a content aggregator in Kampala (Yo! Uganda Limited, Kampala, Uganda), which relayed the automated SMS text messages to the indicated phone number. All SMS text messages, including both incoming and outgoing, were paid for by the study through use of a short code.

### Outcomes Assessment

Study staff recorded the date of clinic return for all participants. Participants who returned within 14 days of the first scheduled message completed an in-person follow-up questionnaire. Research assistants called those who did not return 14 days after the first scheduled message to complete the interview. Questions included whether or not participants received the message, the number of SMS text messages received, and identification of the type of message received.

### Statistical Analyses

We used standard data summarization techniques to describe characteristics for the total cohort and by laboratory result subgroups (normal vs abnormal laboratory result). We assessed for predictors of 3 outcomes of interest: (1) reported receipt of at least one SMS text message, (2) accurate identification of the message format delivered, and (3) whether participants returned to care at the appropriate time, defined as within 7 days of the first SMS text message for those with abnormal results or on the date of the scheduled appointment for those with normal results. For predictors of each outcome, we performed stratified analyses by presence or absence of abnormal test results and assessed for statistically significant relationships with the following baseline characteristics: age, gender, CD4 count result, net household income, educational attainment, ability to read a complete sentence, duration of time required to reach the HIV clinic, whether or not they shared their mobile phone with others in the household, ability to access a sample test message on enrollment (for those with a phone available that day), number of messages sent, cellular network used, and—for those with abnormal results—the type of message sent (direct, PIN, or coded). Crude associations between explanatory variables and outcomes were performed with chi-square testing. We assessed for independent predictors of each outcome by fitting multivariable logistic regression model, including age, gender, CD4 count result, literacy, ability to access a test SMS text message on enrollment, and message type. We assessed for an interaction effect between literacy on enrollment and SMS text message format in 2 separate analyses restricted to either those who were sent (1) direct and PIN messages, or those who were sent (2) direct and coded messages. Although we did not include household income, educational attainment, or number of messages sent because they were not significant in any crude analyses, we performed sensitivity analyses with them added to the multivariable models to assess for negative confounding. All statistical analyses were performed with Stata version 13.1 (StataCorp LP, College Station, TX, USA).

### Ethical Considerations

The study was reviewed and approved by the ethical review committees of the Mbarara University of Science and Technology, Partners Healthcare, and the Ugandan National Council of Science and Technology. The trial was registered at clinicaltrials.gov (NCT01579214).

## Results

We enrolled 385 participants into the trial during the intervention period. The median age of participants was 32 years (IQR 26-39), 65.2% (251/385) were female, more than 60% (240/385, 62.3%) had a primary education or less, median monthly household income was US $80/month (IQR 36-180), and 67.5% (260/385) successfully read a complete sentence on enrollment (Table 1). Nearly half of participants (164/385, 42.6%) shared their mobile phone with others. Of those who had a mobile phone available on the day of enrollment (315/385, 81.8%), approximately three-quarters (247/315, 78.4%) successfully accessed and read the test message. In all, 138 of 385 participants (35.8%) had an abnormal test result. All participants who had a normal result were sent only a single SMS text notification, whereas most with abnormal test results were sent the maximum allowed 7 daily messages (100/138, 72.5%).

**Table 1:** Cohort characteristics.

Cohort characteristics		Total cohort (N=385)	Abnormal result (n=138)	Normal result (n=247)
Age (years), median (IQR)		32 (26-39)	30 (25-38)	34 (26-4)
Gender (female),n (%)		251 (65.2)	75 (54.3)	176 (71.3)
CD4 count, median (IQR)		409 (272-549)	224.5 (110-293)	504 (419-654)
**Estimated household income (US $/month), n (%)**				
	<40	77 (20.0)	20 (14.5)	57 (23.2)
	40-80	81 (21.0)	26 (18.8)	55 (22.3)
	80-180	58 (15.1)	30 (21.7)	28 (11.3)
	>180	69 (17.9)	26 (18.9)	43 (17.4)
	Unknown/unable to estimate	100 (26.9)	36 (26.1)	64 (25.9)
**Educational attainment, n (%)**				
	Less than primary	43 (11.2)	12 (8.7)	31 (12.6)
	Primary	197 (51.2)	71 (51.4)	126 (51.2)
	Secondary	104 (27.0)	37 (26.8)	67 (27.1)
	Beyond secondary	41 (10.6)	18 (13.0)	23 (9.3)
**Successfully read a complete sentence in Runyankole at enrollment visit, n (%)**		260 (67.5)	97 (70.3)	163 (66.0)
Estimate duration of journey to clinic (minutes), median (IQR)		60 (30-120)	42.5 (20-90)	60 (30-120)
Shared mobile phone, n (%)		164 (42.6)	49 (35.5)	115 (46.6)
**Available mobile phone at enrollment visit, n (%)**		315 (81.8)	111 (80.4)	204 (82.6)
	Successfully accessed test message	247 (78.4)	93 (83.8)	154 (75.5)
**Messages sent, n (%)**				
	1	249 (64.6)	2 (1.4)	247 (100)
	2-6	36 (9.4)	36 (26.1)	0
	7	100 (26.0)	100 (72.5)	0
**Cellular network, n (%)**				
	MTN	199 (51.6)	68 (49.3)	131 (54.1)
	Airtel	111 (28.8)	43 (31.6)	68 (28.1)
	Warid	61 (15.8)	23 (16.7)	38 (15.7)
	Other	8 (2.1)	3 (2.2)	5 (2.1)
**Message type, n (%)**				
	Normal	247 (64.2)		247 (100)
	Direct	46 (11.9)	46 (33.3)	0
	PIN	48 (12.5)	49 (35.5)	0
	Coded	43 (11.2)	43 (31.2)	0
**Study outcomes, n/N (%)**				
	Received at least 1 SMS text message	250/346 (72.3)	111/138 (80.4)	139/208 (66.8)
	Accurately identified transmitted SMS text format	219/250 (87.6)	87/111 (78.4)	132/139 (95.0)
	Returned to clinic based on SMS text instructions	234/385 (60.8)	78/138 (56.5)	156/247 (62.8)

For the entire cohort, 72.3% (250/346) reported successful receipt of a message, 87.8% (219/250) of whom correctly identified the message format and 60.0% (231/385) returned to clinic at the requested time. For participants with abnormal tests results, these proportions were 80.4% (111/138), 78.4% (87/111), and 56.5% (78/138), respectively. Although there were no independent predictors in multivariable models for reported receipt of at least one SMS text message, the ability to read a sentence (85%, 82/97 vs 71%, 27/38; *P*=.07) and ability to access and read a test message on enrollment (83%, 77/93 vs 61%, 11/18; *P*=.04) were most closely associated in crude analyses (Table 2). The ability to read a complete sentence on enrollment was independently associated with accurate identification of the message sent (AOR 4.54, 95% CI 1.42-14.47, *P*=.01) and return to clinic within 7 days of the first transmitted SMS text message (AOR 3.81, 95% CI 1.61-9.03, *P*=.002). An ability to access an SMS text message on enrollment was also independently associated with returning to clinic within 7 days of abnormal SMS text notification (AOR 4.90, 95% CI 1.06-22.61, *P*=.04).

**Figure 2:**
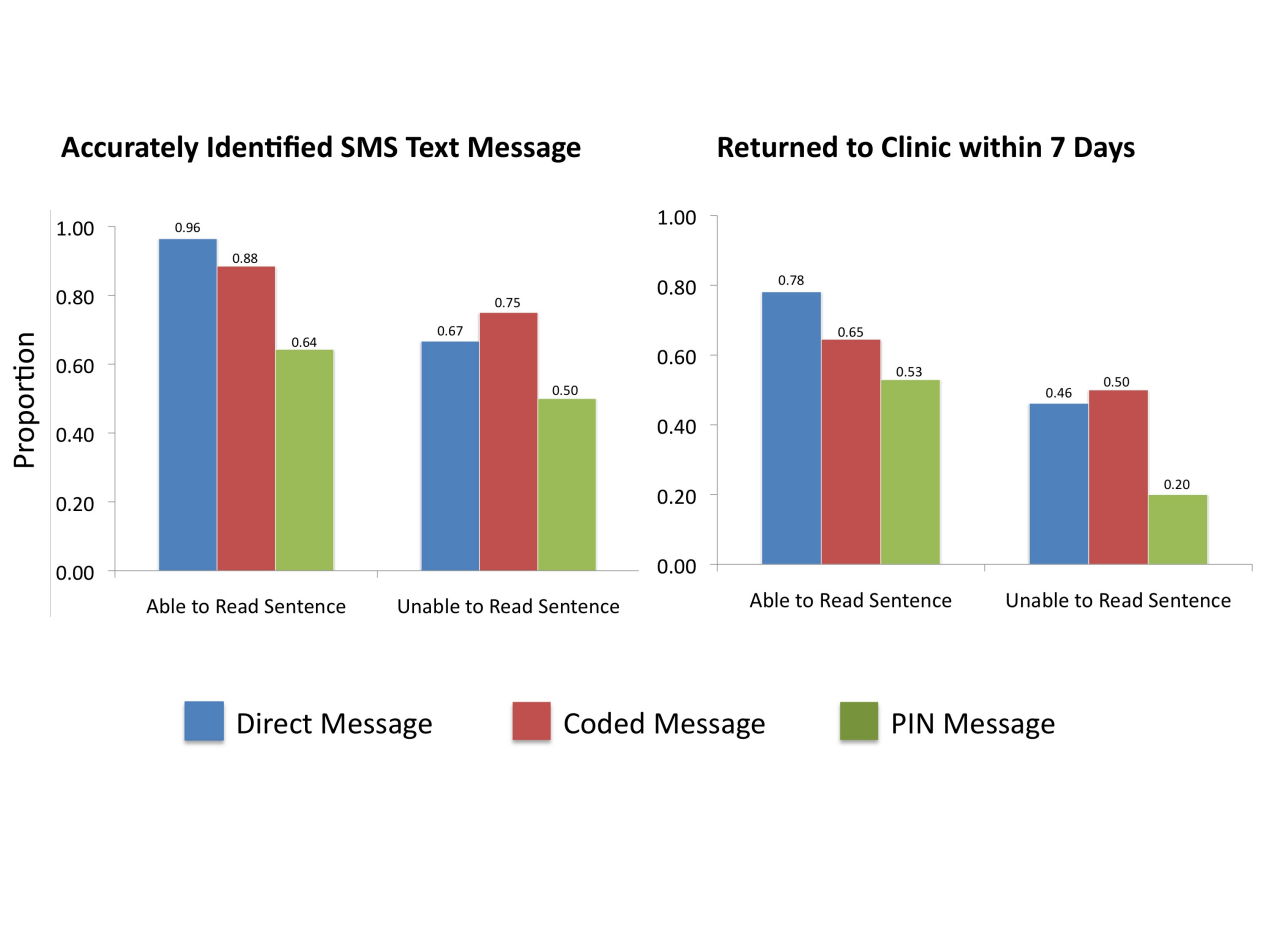
Outcomes by SMS text message format and literacy.

In addition to literacy and mobile phone familiarity, SMS text message format was an important predictor of outcomes for participants with abnormal laboratory results. Compared to receipt of a direct message, those with a PIN-protected message were significantly less likely to identify the message sent (AOR 0.11, 95% CI 0.03-0.44, *P*=.002) or return to clinic within 7 days (AOR 0.26, 95% CI 0.10-0.66, *P*=.005) (Table 3). The odds of SMS text message identification and return to clinic were nominally decreased with receipt of a coded versus direct message, but these associations were not statistically significant (Table 3). In restricted analyses comparing either direct versus PIN or direct versus coded messages, we found no statistically significant interaction terms between literacy and message type, suggesting that both literacy and message type were independent predictors of outcomes (Figure 2). Age, gender, household income, educational attainment, and number of messages sent were not associated with any outcomes for participants with abnormal laboratory results in crude analyses. In sensitivity analyses with each of these variables added to the models, we found no substantial differences in our estimates of association with literacy or message format. Lastly, outcomes for those with abnormal test results did not vary meaningfully by telecommunication network used.

We found similar results for predictors of outcomes for participants with normal results (Table 4). Report of receiving an SMS text message was lower for those with normal test results (66.8%, 139/208 vs 80%, 111/138; *P*=.006), whereas those who did receive a message were more likely to appropriately identify the message received (94.9%, 132/139 vs 78.4%, 87/111; *P*<.001). Both an ability to read a sentence on enrollment (74%, 104/140 vs 51%, 35/68; *P*=.001) and the ability to access a test message on enrollment (74%, 96/130 vs 58%, 23/40; *P*=.049) were associated with receipt of a message. Aside from network type, which was associated with accurate identification of a message, we found no other significant predictors of outcomes for those with normal laboratory results.

**Table 2:** Outcomes for participants with an abnormal laboratory result.

Characteristic		Reported text receipt, n/N (%)	*P*	Accurate message identification, n/N (%)	*P*	Appropriate return to clinic, n/N (%)	*P*
All abnormal result participants		111/138 (80.4)	n/a	87/111 (78.4)	n/a	78/138 (56.5)	n/a
**Message type**			.81		.004		.045
	Direct	38/46 (83)		34/38 (89)		31/46 (67)	
	PIN	38/49 (78)		23/38 (60)		21/49 (43)	
	Coded	35/43 (81)		30/35 (86)		26/43 (60)	
**Age (years)**			.64		.50		.98
	≤25	26/35 (74)		22/26 (85)		20/35 (57)	
	26-32	30/38 (79)		23/30 (77)		22/28 (58)	
	33-39	25/30 (83)		21/25 (84)		16/30 (53)	
	≥40	30/35 (86)		21/30 (70)		20/35 (57)	
**Gender**			.57		.73		.89
	Female	59/75 (79)		40/52 (77)		42/75 (56)	
	Male	52/63 (83)		47/59 (80)		36/63 (57)	
**CD4 result**			.78		.65		.04
	≤100	27/34 (79)		22/27 (81)		25/34 (73)	
	101-350	84/103 (82)		65/84 (77)		43/103 (51)	
**Estimated household income (US $/month)**			.13		.62		.66
	<40	14/20 (70)		12/14 (86)		13/20 (65)	
	40-80	19/26 (73)		13 /19 (68)		13/26 (50)	
	80-180	22/30 (73)		16/22 (73)		15/30 (50)	
	>180	24/26 (92)		19/24 (79)		17/26 (65)	
	Unknown/unable to estimate	32/36 (89)		27/32 (84)		20/36 (56)	
**Educational attainment**			.22		.11		.30
	Less than primary	7/12 (58)		4/7 (57)		4/12 (33)	
	Any primary	59/71 (83)		43/59 (73)		40/71 (56)	
	Any secondary	31/37 (84)		28/31 (90)		24/37 (65)	
	More than secondary	14/18 (78)		12/14 (86)		10/18 (56)	
**Literacy on enrollment**			.07		.03		.003
	Cannot read a complete sentence	27/38 (71)		17/27 (63)		14/38 (37)	
	Reads all of a sentence	82/97 (85)		68/82 (83)		63/97 (65)	
**Transportation time to clinic**			.21		.32		.32
	<30 minutes	30/38 (79)		25/30 (83)		20/38 (53)	
	30-59 minutes	28/37 (76)		19/28 (68)		20/37 (54)	
	60-119 minutes	29/31 (94)		25/29 (86)		22/31 (71)	
	≥129 minutes	24/32 (75)		18/24 (75)		16/32 (50)	
**Share mobile phone with household**			.79		.87		.41
	No	71/89 (80)		56/71 (79)		48/89 (54)	
	Yes	40/49 (82)		31/40 (78)		30/49 (61)	
**Accessed sample SMS text message on enrollment**			.04		.62		.03
	No	11/18 (61)		8/11 (73)		6/18 (33)	
	Yes	77/93 (83)		61/77 (79)		57/93 (61)	
**Messages sent**			.45		.70		.84
	1-6	29/38 (76)		22/29 (76)		22/38 (58)	
	7	82/100 (82)		65/82 (79)		56/100 (56)	
**Cellular network**			.67		.07		.93
	MTN	56/68 (83)		44/56 (79)		38/68 (56)	
	Airtel	35/43 (81)		24/35 (69)		23/43 (53)	
	Warid	17/23 (74)		17/17 (100)		14/23 (61)	
	Other	3/3 (100)		2/3 (67)		2/3 (67)	

**Table 3:** Multivariable logistic regression models among participants with abnormal test results for predictors of successful receipt of at least one SMS text message, accurate identification of the transmitted SMS text message, and return to clinic within 7 days of message transmission.

Characteristic		Reported SMS receipt		Accurate SMS identification		Return to clinic <7 days	
		AOR (95% CI)	*P*	AOR (95% CI)	*P*	AOR (95% CI)	*P*
**Age (years)**							
	<26	reference		reference		reference	
	26-32	0.97 (0.30-3.13)	.97	0.33 (0.07-1.60)	.17	0.71 (0.24-2.07)	.53
	33-39	1.43 (0.40-5.16)	.59	0.98 (0.18-5.27)	.99	0.72 (0.23-2.19)	.56
	≥40	1.49 (0.41-5.45)	.54	0.24 (0.05-1.19)	.08	0.66 (0.22-1.95)	.45
Gender (female)		0.95 (0.38-2.37)	.92	1.30 (0.44-3.83)	.63	1.15 (0.52-2.52)	.73
**CD4 result**							
	≤100	reference		reference		reference	
	101-350	1.08 (0.39-2.96)	.89	0.51 (0.13-1.96)	.33	0.28 (0.11-0.75)	.01
Read a complete sentence on enrollment		2.14 (0.85-5.39)	.11	4.54 (1.42-14.47)	.01	3.81 (1.61-9.03)	.002
Accessed sample SMS text on enrollment^a^		3.05 (0.76-12.21)	.12	0.63 (0.08-4.68)	.65	4.90 (1.06-22.61)	.04
**Randomized SMS text format**							
	Direct	reference		reference		reference	
	PIN	0.76 (0.27-2.17)	.61	0.11 (0.03-0.44)	.002	0.26 (0.10-0.66)	.005
	Coded	1.00 (0.31-3.20)	.99	0.38 (0.08-1.80)	.22	0.58 (0.22-1.55)	.28

^a^ Restricted to participants with an available mobile phone on enrollment.

**Table 4:** Outcomes for participants with a normal laboratory result.

Characteristic		Reported SMS receipt, n/N (%)	*P*	Accurate message identification, n/N (%)	*P*	Appropriate return to clinic, n/N (%)	*P*
All normal test result participants		139/208 (66.8)	n/a	132/139 (95.0)	n/a	156/247 (63.2)	n/a
**Age (years)**			.75		.26		.61
	≤25	26/40 (65)		23/26 (88)		34/48 (71)	
	26-32	31/42 (74)		31/31 (100)		32/50 (64)	
	33-39	37/56 (66)		35/37 (95)		39/66 (59)	
	≥40	45/70 (64)		43/45 (96)		51/83 (61)	
**Gender**			.14		.28		.26
	Female	93/146 (64)		87/93 (94)		115/176 (65)	
	Male	46/62 (74)		45/46 (98)		41/71 (58)	
**Estimated household income (US $/month)**			.14		.46		.85
	<40	29/48 (60)		26/29 (90)		37/57 (65)	
	40-80	34/45 (76)		33/34 (97)		34/55 (62)	
	80-180	16/27 (59)		15/16 (94)		17/28 (61)	
	>180	28/35 (80)		28/28 (100)		30/43 (70)	
	Unknown/unable to estimate	32/53 (60)		30/32 (94)		38/64 (59)	
**Educational attainment**			.14		.29		.11
	Less than primary	15/27 (56)		13/15 (87)		22/31 (71)	
	Any primary	64/101 (63)		62/64 (97)		77/126 (61)	
	Any secondary	44/61 (72)		41/44 (93)		38/67 (57)	
	More than secondary	16/19 (84)		16/16 (100)		19/23 (83)	
**Literacy on enrollment**			.001		.27		.27
	Cannot read a complete sentence	35/68 (51)		32/35 (91)		57/84 (68)	
	Reads all of a sentence	104/140 (74)		100/104 (96)		99/163 (61)	
**Transportation time to clinic**			.36		.95		.37
	<30 minutes	32/44 (73)		30/32 (94)		34/48 (71)	
	30-59 minutes	32/47 (68)		31/32 (97)		37/54 (69)	
	60-119 minutes	38/65 (58)		36/38 (95)		45/77 (58)	
	≥129 minutes	37/52 (71)		35/57 (95)		40/68 (59)	
**Share mobile phone with household**					.34		.67
	No	75/116 (75)	.46	70/75 (93)		85/132 (64)	
	Yes	64/92 (70)		62/64 (97)		71/115 (62)	
**Accessed sample SMS text on enrollment**			.049		.52		.17
	No	23/40 (58)		21/23 (91)		27/50 (54)	
	Yes	96/130 (74)		91/96 (95)		100/154 (65)	
**Cellular network**			.14		<.001		.99
	MTN	84/113 (74)		82/84 (98)		85/131 (65)	
	Airtel	35/58 (60)		35/35 (100)		43/68 (63)	
	Warid	18/30 (60)		13/18 (72)		24/38 (63)	
	Other	2/2 (100)		2/2 (100)		3/5 (60)	

## Discussion

### Principal Findings

Among PLWH in rural Uganda, confirmed literacy at the time of enrollment, a demonstrated ability to access a sample SMS text message, and absence of a PIN-code protector were robust predictors of receipt, identification, and appropriate response to an SMS text–based laboratory result messaging app. Specifically, PIN-protected messages were poorly accessed and reduced odds of message identification and appropriate clinic return. However, coded messages, which obviate the need for literacy, were as effective as direct messages and might augment privacy. We found no associations between age, gender, educational attainment, household income, or number of messages sent on any outcomes. Many studies have explored the acceptability of SMS text message interventions among PWLH in resource-limited settings, but our study is the first to our knowledge to directly assess the impact of literacy and technology experience on process and clinical outcomes, and demonstrates the importance of such features for mHealth interventions in these settings.

Prior work, largely among high-literacy users in resource-rich settings, has demonstrated that 2 major constructs—perceived ease of use and perceived usefulness—are important predictors of intention to use and use of health technology [9]. Two conceptual models of technology acceptance, the technology acceptance model and the unified theory of technology acceptance and use of technology, have validated the importance of additional mediators, including subjective norms, output quality, and technology experience [23-25]. Studies among patient end users in resource-limited settings are relatively scarce, largely restricted to preintervention assessments, and have demonstrated high potential for acceptance [11,12,14,26,27]. Our study demonstrates that among a patient population with a near 100% acceptability for SMS text-based mHealth interventions [12], rates of SMS text message receipt (73%), accurate SMS text message identification (88%), and appropriate response to SMS text request (63%) were modest. Although we do not report results of ease of use or perceived ease of use directly, our findings that literacy, mobile phone experience, and non-PIN-protected messages all strongly support predictive roles for ease of use as a dominant role for successful technology uptake. Other postintervention assessments in resource-limited settings have demonstrated similar effects. A study assessing technology acceptance of self-service health kiosks in South Africa found that ease of use was the strongest predictor and most correlated with technology anxiety and self-efficacy [28]. A second study from South Africa evaluating acceptability of a mobile phone-assisted personal interview to augment face-to-face maternal health data collection among health care workers found that both ease of use and perceived usefulness improved after a training session [15]. A study of SMS text-based antiretroviral adherence reminders for caregivers of pediatric patients demonstrated low uptake of PIN-protected messages [8]. We attempted to mitigate the complexity of PIN-based messages by allowing participant-selected codes and giving participants a form with the code, but we found similarly poor results for PIN-based messages. Finally, an analysis to determine predictors of response to an SMS text-based ART adherence reminder intervention demonstrated that higher educational attainment predicted improved adherence [29].

Because an important minority of patients in a prestudy interview reported confidentiality concerns about receiving clinic-related information by SMS text message [12], we attempted to assess various message formats to evaluate trade-offs between privacy and clarity. Whereas PIN code-protected messages were negatively associated with successful outcomes in our study, we were able to demonstrate the feasibility of protecting patient confidentiality through use of non-text messages without need for PIN-code protection. Participants who received a message stating “ABCDEFG” had similar rates of message receipt, identification, and early clinic return times as those who received a full instructional text message. Although this method shows promise, it is limited to transmission of qualitative messages (eg, “yes” or “no” information). Prior studies of mHealth interventions have attempted alternative strategies including not mentioning the words “HIV” or “ART” [30] or using nonspecific greetings [31]. One prior study, which did specifically use the term “medication” in an SMS text-based adherence reminder, found no effect of the intervention [32].

Our results have important implications for future mHealth interventions targeted to low-literacy end users. First, text messaging was broadly successful in a rural, resource-limited population with limited education, suggesting that age, gender, educational attainment, and income should not be used as screening criteria for SMS text message interventions. Second, thorough assessments of end-user written literacy and technology experience should be made before and during implementation design. Third, we found that in-person confirmation of mobile phone competency was highly predictive and should be considered for future similar interventions where possible. Fourth, we demonstrate that coded messages can have similar efficacy as text messages, while maintaining confidentiality. Importantly, our study involved transmitting qualitative information (ie, normal or abnormal laboratory results). More complex instructional messages or quantified information will present additional challenges that should be explored in future studies through use of pictorial or other symbolic message formats. Finally, we observed increased rates of reported message receipt among those in the abnormal results group who mostly received 7 daily messages (median 7, IQR 5-7) compared to those in the normal results group who only received a single message, suggesting that repeated messages might increase successful transmission. In contrast, prior work has showed that weekly messages might be modestly preferably to daily messages [30]. However, our study involved only a single notification, as opposed to prior adherence studies that transmitted SMS text message reminders for up to a year.

### Limitations

Our study was conducted at a single clinical site with a highly impoverished and low-literacy population. Although this limits the generalizability of our study, it also adds important data about a study population in a low resource setting, which is often the target of mHealth interventions. Our study would have also benefited from evaluation of additional message formats. For example, interactive voice response or direct voice call groups would have added important comparative information; however, they might have challenged the scalability of the intervention.

### Future Work

We are pursuing further activities to build on these results. Specifically, we are conducting postintervention qualitative interviews to collect in-depth accounts about ease of use and usefulness of the information, as well as barriers and promoters of uptake of the intervention. Lastly, we have partnered with the clinic data managers and faculty members in computer science at the Mbarara University of Science and Technology to implement an SMS text–based reminder and results messaging system clinic-wide. We are planning a second evaluation following implementation to learn about large-scale effectiveness and scalability.

### Conclusions

In summary, we demonstrate that end-user characteristics, particularly literacy and technology experience, are important predictors of an mHealth intervention for PLWH in rural Uganda. We also demonstrate that, although PIN code–protected messages decrease the efficacy of SMS text message information delivery in this population, privacy can be maintained through coded messaging. Future SMS text–based interventions for low-literacy users in similar settings should consider these factors in design and implementation of mHealth interventions. Further evaluation of technology acceptance in this population and similar ones is needed if the potential of mHealth in sub-Saharan Africa is to be realized.

## Abbreviations

API: application program interface
ART: antiretroviral therapy
HIV: human immunodeficiency virus
PIN: personal identification number
PLWH: people living with HIV
SMS: short message service
